# The role of 18F-FDG PET/CT in spinal infections: A comprehensive diagnostic tool

**DOI:** 10.1016/j.bas.2026.106106

**Published:** 2026-05-22

**Authors:** Raimunde Liang, Solveig Hirsch, Susan Notohamiprodjo, Sebastian Ille, Eugen Ursu, Leonhard Reichel, Maria Wostrack, Wolfgang A. Weber, Bernhard Meyer, Ann Kathrin Joerger

**Affiliations:** a Department of Neurosurgery, TUM University Hospital, TUM School of Medicine and Health, Technical University of Munich (TUM), Munich, Germany; b Department of Nuclear Medicine, TUM University Hospital, TUM School of Medicine and Health, Bavarian Cancer Research Center (BZKF), Technical University of Munich (TUM), Munich, Germany; c Department of Neurosurgery, Heidelberg University Hospital, Heidelberg, Germany; d New Affiliation: Department of Neurosurgery, BG Trauma Center Murnau, Murnau, Germany

**Keywords:** Spondylodiscitis, Spinal infection, Positron emission tomography

## Abstract

**Introduction:**

Spondylodiscitis poses diagnostic challenges due to nonspecific symptoms and limitations of conventional imaging modalities. Moreover, causative infection foci often remain unidentified. Early and accurate diagnosis and treatment of the infectious focus are essential to prevent severe complications. ^18^F-fluorodeoxyglucose positron emission tomography (FDG-PET) has emerged as complementary tool to MRI, enabling detection of metabolically active infectious processes.

**Research question:**

This study evaluates the diagnostic accuracy of FDG-PET/CT in patients with suspected spondylodiscitis and its ability to identify additional infectious foci.

**Material and methods:**

A retrospective analysis included 191 patients who underwent FDG-PET/CT for suspected spondylodiscitis between 01.01.2016 and 31.12.2023. PET findings were compared with MRI, pathological, and microbiological and clinical data.

**Results:**

Spinal infection was confirmed in 160 of 191 patients. In patients with inconclusive MRI findings (n = 46), FDG-PET/CT led to diagnostic reclassification in 21 cases (45.7%). In the subgroup analyzed for diagnostic accuracy (n = 56), sensitivity was 56% for FDG-PET/CT and 62% for MRI, increasing to 74% when both modalities were combined (p = 0.002). Specificity was 96% for FDG-PET/CT and 86% for MRI. FDG-PET/CT identified 23 previously unknown extraspinal infectious foci and confirmed 23 suspected foci, resulting in detection of infectious sources in 73% of patients. Infectious foci were detected more frequently in cervical infections (33.3% vs. 9.5%, p = 0.001). Patients undergoing FDG-PET/CT showed higher clinical resolution rates at follow-up (p = 0.045).

**Discussion and conclusion:**

FDG-PET/CT provides complementary value in suspected spondylodiscitis, particularly in cases with inconclusive MRI and for detecting additional infectious foci, supporting its role in multimodal diagnostic strategies.

## Highlights

• FDG-PET/CT reclassified nearly half of equivocal MRI findings.
• Combined PET/CT and MRI improved sensitivity for spondylodiscitis.
• Cervical infection was linked to a threefold higher PET/CT focus yield.

## 1 Introduction

Spondylodiscitis is an increasingly recognized cause of morbidity and mortality, particularly among elderly patients (Gouliouris et al., 2010). Early and accurate diagnosis is essential to prevent complications such as epidural, paravertebral, or psoas abscesses, spinal instability, neurologic deficits, and septicemia; however, nonspecific symptoms often lead to a delay in diagnosis and treatment.

Spondylodiscitis can arise through several pathways, most commonly via hematogenous spread from a distant infectious focus. Identifying the source of infection is crucial, as its eradication is essential for effective and targeted treatment (Lener et al., 2018; Widdrington et al., 2018). However, in approximately 17% to 46% of cases, the underlying infectious source remains unidentified (Widdrington et al., 2018; Pigrau et al., 2015).

Contrast-enhanced magnetic resonance imaging (MRI) is currently the gold standard for the diagnosis of spondylodiscitis due to its high sensitivity and specificity of over 90% (Sans et al., 2012). Nevertheless, MRI may yield inconclusive results in early stages of infection before anatomical changes become evident and patients with metal implants due to artifact formation. Additionally, MRI findings can sometimes resemble degenerative spinal changes, making it difficult to distinguish these from spondylodiscitis (Dunbar et al., 2010; Carragee, 1997).

Fluorine-18 2′-deoxy-2-fluoro-D-glucose positron emission tomography computed tomography (FDG-PET/CT) has emerged as an increasingly valuable imaging modality in the diagnostic workup of spinal infections due to its high sensitivity and specificity for detecting inflammatory activity (Schmitz et al., 2001; Kouijzer et al., 2018). FDG-PET/CT can be used in patients with contraindications for MRI, such as implanted pacemakers, and provides diagnostic image quality even in the presence of metallic hardware, where MRI is often limited by artifacts. Additionally, FDG-PET/CT allows for fast whole-body imaging, enabling the detection of distant infectious foci in addition to evaluation of the spine. Despite its benefits, FDG-PET/CT remains underutilized, primarily due to cost and limited availability (Abikhzer et al., 2025).

Most existing studies evaluating FDG-PET/CT in spondylodiscitis are based on small cohorts, with only few meta-analyses and focus primarily on local diagnostic accuracy in comparison to MRI (Smids et al., 2017; Altini et al., 2020). This study aims to evaluate the clinical utility of FDG-PET/CT in the diagnostic workup of patients with spinal infections, while also considering the broader diagnostic value of PET/CT in identifying additional infectious foci beyond the spine.

## 2 Material and methods

### 2.1 Patient cohort

We conducted a retrospective analysis at a high-volume Level I spine surgery center, including all patients treated for suspected or confirmed spinal infections between January 1, 2016, and December 31, 2023. Patients were identified by querying the institutional database using ICD-10 codes associated with spinal infections, yielding a total screening cohort of 438 patients. Within this cohort, 191 (44%) patients underwent FDG-PET/CT imaging, while the remaining 247 patients were managed without PET/CT.

Clinical data were extracted through systematic review of medical records, imaging studies, laboratory parameters, microbiological and histopathological findings, and surgical reports.

### 2.2 Diagnostic and treatment

The diagnosis of spondylodiscitis, spinal empyema, or infection-related implant loosening is established based on a combination of clinical presentation (typically therapy-refractory back pain), characteristic findings on CT and contrast-enhanced MRI, and microbiological confirmation through blood cultures or CT-guided biopsy. The date of hospital admission was defined as the date of diagnosis. As part of the diagnostic workup, all patients included in our cohort underwent FDG-PET/CT. Whole-spine MRI represented the standard first-line imaging modality and was available for the majority of patients. However, not all patients were eligible for MRI due to contraindications, such as non–MRI-compatible cardiac pacemakers or implantable cardioverter-defibrillators; in these cases, FDG-PET/CT was used as the primary diagnostic modality.

In the early study period, FDG-PET/CT was reserved for selected cases, primarily for focus detection when conventional diagnostics failed to identify an infectious source or when MRI findings were inconclusive, particularly in the setting of extensive spinal instrumentation, or when contraindications to MRI were present, as outlined above. From 2020 onward, FDG-PET/CT became a standard component of the diagnostic workup for infectious focus detection, except in patients who were too clinically unstable to undergo this relatively time-consuming examination. Surgical treatment was performed in nearly all cases, except in patients with excessive perioperative risk or in those who died before surgery could be initiated. Standard surgical management of spondylodiscitis included posterior instrumentation using pedicle screws, debridement of the infected disc space via a dorsal or anterolateral approach, and interbody fusion with cage placement. Patients with isolated spinal empyema underwent surgical evacuation via a dorsal approach. Low-grade infection-related implant loosening was managed through implant removal, followed by antibiotic therapy and later reassessment for potential re-instrumentation.

Empirical broad-spectrum intravenous antibiotics, typically initiated after collection of biopsy material or blood cultures, were administered promptly. Once microbiological results became available, antibiotic regimens were tailored accordingly. All patients received at least two weeks of intravenous antibiotic therapy, followed by an oral regimen to complete a total of 12 weeks of antimicrobial treatment. Patients underwent routine clinical and laboratory follow-up toward the end of antibiotic therapy, and clinical resolution was defined by absence of infection-related symptoms in combination with normalization or marked improvement of inflammatory markers.

### 2.3 FDG-PET/CT examination

All patients fasted for at least 4 h prior to the ^18^F-FDG tracer injection. Blood glucose levels were required to be less than 140 mg/dL during a period of approximately 60 min before the administration of ^18^F-FDG (3 MBq/kg). Diagnostic CT imaging was performed in the portal venous phase 80 s after intravenous injection of contrast agent [Imeron 300] (1.5 mL/kg body weight, max. 120 mL) followed by the PET imaging approximately 60 min after ^18^F-FDG injection. The axial field of view ranged at least from the pelvic floor to the top of the head. The extremities were additionally imaged when there a focus was expected in this area.

### 2.4 Statistical analysis

Statistical analyses were performed using GraphPad Prism Version 9.4.1 (San Diego, California, U.S.A.). and internally developed Python scripts utilizing the SciPy library. Categorical data was compared using the chi-square test as needed. For paired nominal data, McNemar's test was applied. Mean values were compared using the independent-samples *t*-test. All tests were performed two-sided, and a p-value <0.05 was considered significant.

### 2.5 Ethical considerations

This study was conducted in accordance with the ethical principles outlined in the Declaration of Helsinki. Our local ethics committee approved the study before data collection (registration number 2023-324_1-S-SB). Given the retrospective nature of our research, our local ethics committee waived informed consent.

## 3 Results

### 3.1 Patient cohort

A total of 191 patients who underwent 209 FDG-PET/CT-scans for suspected spinal infection were included in the study. Of these, twelve patients underwent two examinations and three patients underwent three examinations. The mean age was 69 ( ± 13) years, and most patients were male (n = 126, 66%). Detailed clinical characteristics are shown in Table 1.

**Table 1:** **Patient cohort.** Baseline demographic and clinical characteristics of the study cohort.

Variable	Overall FDG-PET/CT cohort (n = 191)	Confirmed spinal infection (n = 160)
Mean Age, years (STD)	69 ( ± 13)	70 ( ± 12)
Male/Female (%)	126/65 (66/34)	108/52 (68/32)
MRI performed	179	153
Mean CRP, mg/dl (STD)	10 ( ± 9)	10 ( ± 9)
Localisation	102	87
Lumbar	28	22
Thoracic	20	19
Cervical	37	32
Multiregional	4	-
Paravertebral		

In 160 patients, a spinal infection was subsequently confirmed, comprising 137 cases of spondylodiscitis, seven cases of isolated spinal empyema, four cases of paravertebral abscess, five cases of infection associated implant loosening and seven of postoperative wound infection. Among these 160 patients, the lumbar spine was the most frequently affected region (87 patients), followed by the thoracic spine (22 patients) and the cervical spine (19 patients). In 31 patients, two spinal regions were affected (18 patients lumbar and thoracic, seven patients lumbar and cervical, six patients thoracic and cervical), and in one patient, all three spinal regions were involved.

In the remaining 31 patients FDG PET/CT scans were conducted in cases in which infection was not subsequently confirmed (n = 30) and/or for follow-up of treated spondylodiscitis (n = 2) with one patients overlapping between these categories.

In addition, a separate cohort of 247 patients with clinically confirmed spinal infection who did not undergo FDG-PET/CT during the same study period was identified for comparative outcome analyses.

### 3.2 Indication for FDG-PET/CT imaging

**Fig. 1:**
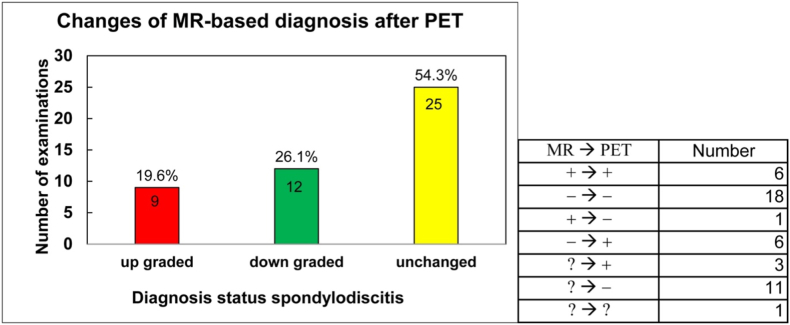
**Impact of FDG-PET/CT on MRI-based diagnosis in patients with inconclusive MRI findings.** Diagnostic status was classified as upgraded (MRI negative or indeterminate to FDG-PET/CT positive), downgraded (MRI positive or indeterminate to FDG-PET/CT negative), or unchanged after FDG-PET/CT. Absolute numbers and corresponding percentages are shown.

Among the included patients, FDG-PET/CT was performed to identify the infectious focus in 175 cases (Table 2). In 46 cases FDG-PET/CT was conducted due to inconclusive MRI findings, to confirm or exclude spondylodiscitis. In this subgroup with equivocal MRI results, FDG-PET/CT led to a change in the initial MRI-based diagnostic assessment in a substantial proportion of patients (Fig. 1). Following FDG-PET/CT, the diagnosis was “upgraded” to spondylodiscitis in nine cases (19.6%) and “downgraded” (spondylodiscitis ruled out) in twelve cases (26.1%), whereas MRI-based suspicion remained unchanged in 25 cases (54.3%) (Fig. 1). Overall, FDG-PET/CT resulted in diagnostic reclassification in 21 of 46 patients (45.7%). In ten patients, FDG-PET/CT served as an alternative to MRI, either due to contraindications or patient intolerance. In six additional cases, FDG-PET/CT was performed as a follow-up investigation to assess the resolution or persistence of previously diagnosed spondylodiscitis.

**Table 2:** **Indication for FDG-PET/CT imaging.** Data are shown for the entire cohort and for the subgroup with clinically confirmed spinal infection. Clinically confirmed spinal infection was defined based on clinical presentation supported by intraoperative and/or microbiological and/or histopathological findings.

Indication	Overall FDG-PET/CT cohort (n = 191)	Confirmed spinal infection (n = 160)
Focus identification	175	152
Inconclusive MRI	46	10
Substitute for MRI	10	7
Follow up imaging	6	-

### 3.3 FDG-PET/CT for identifying extraspinal sources of infection

Among the 160 patients with subsequently confirmed spinal infection, FDG-PET/CT was performed in 152 cases to identify a potential infectious focus (Table 2). In this cohort, before FDG-PET/CT was performed, the presumed etiology of spondylodiscitis was already known in 61 patients due to prior spinal surgery, and in 48 patients an extraspinal infectious focus had been clinically identified. FDG-PET/CT confirmed the clinically identified extraspinal infectious focus in 23 of these cases (Table 3), whereas it missed the infectious focus in nine cases, including one distal skin laceration outside the field of view, five dental infections, and three cases of endocarditis.

**Table 3:** **Findings from FDG-PET/CT Imaging.** The table summarizes the results in absolute numbers for the subgroup with confirmed spinal infection (n = 160).

	number of patients (n = 160)
Identification of additional infectious sites	23
Confirmation of known extraspinal infectious foci	23
Missed infectious foci	9
Clinically non-specific FDG uptake	16

FDG uptake suspicious for an extraspinal infectious focus was uptake observed in 53 cases. Of these, 23 represented previously unknown infectious foci, predominantly located in the joints. Among these, eight represented the first identifiable focus of infection, whereas 15 constituted additional foci in patients with an already known infectious site. In contrast, nine cases showed increased FDG uptake that was initially suspected to indicate infection but was ultimately classified as clinically non-specific, most commonly involving joints, teeth, or the gastrointestinal tract. Overall, a clinically confirmed focus of infection (spinal or extraspinal) was described in 117 patients (73%). Among the 23 patients with previously unknown foci, FDG-PET/CT findings led to removal or surgical treatment of the infectious focus in 14 patients, such as extraction of an infected tooth or drainage or debridement of an infected joint or abscess. In two additional patients, detection of an extraspinal focus resulted in prolongation of intravenous antibiotic therapy. In the remaining nine patients, the newly detected foci did not result in a separately documented change in management beyond the antibiotic treatment already indicated for spinal infection.

Notably, FDG-PET/CT identified an infectious focus significantly more often in patients with cervical spine infection than in those with non-cervical involvement (33.3% vs. 9.5%, p = 0.001), corresponding to an approximately threefold higher detection rate.

### 3.4 MRI versus FDG-PET/CT for detection and exclusion of spondylodiscitis

For comparative analysis of MRI and FDG-PET/CT, only the 56 patients who underwent FDG-PET/CT prior to spinal surgery were included to avoid confounding effects of early postoperative inflammatory changes. Among these patients, spondylodiscitis was clinically confirmed in 34 cases and excluded in 22 cases. Among the 34 patients with confirmed spondylodiscitis, FDG-PET/CT was positive in 19 cases, while MRI detected spinal infection in 21 cases (Table 4 a). FDG-PET/CT additionally identified four cases of spondylodiscitis missed by MRI; conversely, MRI detected six cases not identified by FDG-PET/CT. This resulted in a sensitivity of 56% for FDG-PET/CT and 62% for MRI. When MRI and FDG-PET/CT findings were combined, overall sensitivity increased to 74%. McNemar's test showed no statistically significant difference between MRI and FDG-PET/CT in detecting confirmed spondylodiscitis (p = 0.75). However, the combination of FDG-PET/CT was significantly more sensitive than MR alone (p = 0.002).

**Table 4:** **Comparison of MRI and FDG-PET/CT in the detection of spondylodiscitis**.a) Results of MRI and FDG-PET/CT imaging in patients with confirmed spondylodiscitis.b) Results of MRI and FDG-PET/CT imaging in patients with exclusion of spondylodiscitis.

a)			
	MRI		
PET	positive	negative	
positive	15	4	19
negative	6	9	15
	21	13	34

b)			
	MRI		
PET	positive	negative	
positive	1	0	1
negative	2	19	21
	3	19	22

Among the 22 patients in whom spondylodiscitis was clinically ruled out, FDG-PET/CT was negative in 21 cases, yielding a specificity of 96%, whereas MRI was negative in 19 cases, corresponding to a specificity of 86%. (Table 4 b). MRI showed false-positive findings in two cases, which were correctly classified as negative by FDG-PET/CT. There was no statistically significant difference between MRI and FDG-PET/CT in the exclusion of spondylodiscitis or the combination of MRI and PET/CT and MRI (McNemar test, p = 0.48).

### 3.5 Impact of FDG-PET/CT on outcomes in spondylodiscitis

Follow-up data were available for 119 patients who underwent FDG-PET/CT imaging. At follow-up assessment 10-12 weeks postoperatively, clinical resolution of spinal infection was observed in 100 patients, whereas eight patients had persistent or recurrent spondylodiscitis, and eleven patients had died. Compared with patients treated for spondylodiscitis at our institution during the same observation period who did not undergo FDG-PET/CT (n = 247), the rate of clinical resolution was significantly higher in the FDG-PET/CT group (Fisher's exact test, p = 0.045).

However, FDG-PET/CT was not associated with a significantly higher detection rate of infectious foci compared with conventional diagnostic workup alone (76.9% in patients undergoing FDG-PET/CT vs. 72.9% in those without FDG-PET/CT; p = 0.34). Furthermore, FDG-PET/CT use was not associated with a statistically significant reduction in in-hospital mortality (6.9% in patients undergoing FDG-PET/CT vs. 10.2% in those without FDG-PET/CT; p = 0.26).

## 4 Discussion

This retrospective analysis assesses the clinical utility of FDG-PET/CT in the evaluation of suspected or confirmed spinal infections in a large cohort of consecutive patients, strengthening the generalizability and clinical relevance of the findings.

### 4.1 FDG-PET/CT for identifying extraspinal sources of infection

Viewed as a systemic disease rather than a purely localized spinal infection, effective management of spondylodiscitis critically depends on identifying and eradicating the underlying source of infection (Gentile et al., 2019). In our cohort, approximately 30% of patients had a known extraspinal infectious focus prior to FDG-PET/CT. These findings are consistent with published data, reporting identification rates of infectious foci in patients with spondylodiscitis ranging from approximately 25% to 35% (Pigrau et al., 2015; Joerger et al., 2023; Pojskic et al., 2021). FDG-PET/CT identified additional previously undetected clinically relevant infectious foci in 14% of patients of our subgroup with confirmed spinal infection, resulting in an overall detection rate of a potential underlying infectious source of 73%. Importantly, eight of these patients had no identifiable infectious focus prior to PET/CT imaging, highlighting the potential of whole-body metabolic imaging to uncover clinically occult sources of infection that may otherwise remain undetected during standard diagnostic workup. Although not reaching statistical significance, these findings support the role of FDG-PET/CT in corroborating suspected sources of infection and enhancing diagnostic confidence. In line with this, previous studies in critically ill patients have reported identification of inflammatory foci by FDG-PET/CT in up to 92% of cases, with subsequent changes in therapeutic management in approximately half of the patients (Treglia, 2019; van Leer et al., 2025).

Infectious foci were missed in a limited number of cases, most notably dental infections and endocarditis. Moreover, FDG-PET/CT showed limited specificity in certain anatomical regions, particularly the joints, dental structures, and the gastrointestinal tract. Increased tracer uptake in these areas may reflect degenerative, inflammatory, or physiological processes rather than active infection, a limitation that has been well described in the literature (Palestro, 2013; Costelloe and Murphy, 2009; Loffeld and Srbjlin, 2019; Treglia et al., 2014). Although FDG-PET/CT generally demonstrates reliable tracer uptake in acute infections, chronic dental lesions are not consistently visualized, likely due to physiological tracer uptake within the oral mucosa and salivary glands, which may obscure pathological findings (Dijkstra et al., 2020; Arefnia et al., 2024). Likewise, the detection of endocarditis remains challenging because variable physiological myocardial 18F-FDG uptake reduces sensitivity (Kouijzer et al., 2013). Reliable suppression of myocardial FDG uptake requires a ketogenic diet for at least 1 day prior to FDG injection which is not feasible during the workup of patients with acute spinal infections (Ozutemiz et al., 2021).

In our cohort, infectious foci were identified approximately three times more frequently in patients with cervical spine infection compared with non-cervical involvement by FDG-PET/CT imaging. Cervical spondylodiscitis has been described as a more critical form of spinal infection compared with thoracic or lumbar disease (Sung and Kim, 2025). Previous reports have also noted an increased rate of concomitant non-spinal infections in these patients, potentially reflecting a marker of systemic disease severity, possibly related to a higher likelihood of hematogenous dissemination (Urrutia et al., 2013; Kong et al., 2025). Although this observation in our cohort should be interpreted cautiously given the limited subgroup size, it may support the consideration of comprehensive systemic evaluation in patients with cervical involvement. In this context, FDG-PET/CT could be particularly valuable, although prospective studies are needed to further clarify its role in this subgroup.

Overall, the reported diagnostic performance of FDG-PET/CT varies widely depending on the underlying infectious entity, with sensitivities ranging from 61% to 98% and specificities from 52% to 98% (Treglia, 2019). Diagnostic accuracy is lowest for endocarditis and highest for osteomyelitis and prosthetic joint infections. Taken together, these findings highlight that FDG-PET/CT provides optimal value when interpreted in conjunction with clinical data and other imaging modalities within an integrated diagnostic strategy.

### 4.2 MRI versus FDG-PET/CT for detection and exclusion of spondylodiscitis

FDG-PET/CT has been reported to achieve a sensitivity of 86–100% and a specificity of 87–95% for the detection of spinal infection, whereas MRI demonstrates a sensitivity of 82–96% and a specificity of 85–93% (Smids et al., 2017; Ledermann et al., 2003; Prodromou et al., 2014). In patients with contraindications to MRI, FDG-PET/CT therefore represents a valuable alternative due to its high diagnostic accuracy in suspected spondylodiscitis. Moreover, FDG-PET/CT is less susceptible to metal-related artifacts and therefore provides more reliable image quality in patients with spinal instrumentation. In this context, higher sensitivity and specificity for FDG-PET/CT compared with MRI have been reported (Follenfant et al., 2019; Segawa et al., 2021; Dauchy et al., 2016). However, the overall sensitivity of FDG-PET/CT for the detection of spondylodiscitis was lower than previously reported in the literature (Smids et al., 2017; Prodromou et al., 2014). This may be explained by differences in study design as our reference standard for the presence of spondylodiscitis was based on comprehensive clinical confirmation, incorporating microbiological, intraoperative, and follow-up data, representing a more stringent definition of spinal infection. In addition, many previously published studies are limited to relatively small sample sizes and more selected patient populations, whereas our cohort reflects a heterogeneous setting, including early-stage or clinically ambiguous cases. Finally, the comparative analysis was restricted to preoperative imaging to avoid confounding by postoperative inflammatory changes. While this approach may introduce some selection bias, it was chosen to improve comparability between imaging modalities and to avoid overestimation of diagnostic accuracy. In particular, in our cohort, FDG-PET/CT had a substantial impact on diagnostic decision-making in patients with MRI findings that were inconclusive. In this diagnostically challenging subgroup, FDG-PET/CT reclassified the initial MRI-based assessment in nearly half of the cases. Moreover, combining FDG-PET/CT with MRI resulted in increased diagnostic sensitivity and specificity compared with either modality alone.

### 4.3 Impact of FDG-PET/CT on outcomes in spondylodiscitis

Notably, patients who underwent FDG-PET/CT had a significantly higher clinical resolution rate than those treated during the same period without FDG-PET/CT imaging. While this association must be interpreted cautiously, given the retrospective study design and potential selection bias, it suggests that FDG-PET/CT-guided diagnostic stratification may contribute to more effective clinical management. Previous studies in other infectious disease settings provide complementary insights. In patients with *Staphylococcus aureus* bacteremia, PET/CT-guided diagnostics have been associated with reduced mortality, largely attributed to improved detection of metastatic infectious foci and subsequent treatment modification (Berrevoets et al., 2017). A large registry-based study by Lang et al. with pooled data also reported an association between FDG-PET/CT use and reduced in-hospital mortality in patients with spondylodiscitis, particularly among elderly patients (Lang et al., 2024). Proposed explanations include earlier diagnosis in early disease stages and improved detection of metastatic or implant-associated infection.

In contrast, we did not observe a significantly higher number of detected infectious foci in our cohort nor a significant reduction in mortality, but rather a higher rate of clinical resolution. This discrepancy may be explained by differences in cohort size and statistical power. It might also indicate that the clinical benefit of FDG-PET/CT in spondylodiscitis is likely multifactorial. Although no significant difference in overall focus detection was demonstrated, FDG-PET/CT identified 23 additional extraspinal infectious foci, including eight patients in whom no infectious source had been detected during the initial clinical workup. Finally, Lang et al. discussed a potential “center effect,” as FDG-PET/CT is typically performed at institutions with established multidisciplinary management pathways and specialized expertise, which per se may confer an outcome and survival advantage independent of the imaging modality itself. As our study was a single-center study and both the FDG-PET/CT cohort and the control group were managed within the same institutional setting using identical multidisciplinary care pathways, a center-related bias is unlikely. Nevertheless, these observations support the hypothesis that improved outcomes associated with FDG-PET/CT may, at least in part, reflect optimized diagnostic workflows and specialized care rather than the imaging modality alone.

### 4.4 Limitations

Several limitations of this study should be acknowledged.

Its retrospective nature and single-center design may limit generalizability.

Furthermore, selection bias cannot be excluded, as the use of FDG-PET/CT changed substantially over the study period. In the early years, it was primarily applied in diagnostically complex cases, whereas in later years it became established as a standard modality for the systematic search for infectious foci. This marked temporal shift in utilization may limit comparability between patients who did and did not undergo PET/CT, particularly with respect to outcome analyses and diagnostic performance, and should be considered when interpreting the results.

Moreover, due to its retrospective design follow-up imaging and outcome assessment were not standardized. The relatively high loss-to-follow-up rate of 26% in the spondylodiscitis cohort warrants consideration. As a tertiary referral center, many patients are transferred back to regional hospitals or to outpatient care after acute treatment for long-term antibiotic therapy, limiting complete long-term outcome documentation in our institutional database. The retrospective design further restricts standardized follow-up and systematic data capture. Although loss to follow-up in this setting likely reflects structural factors rather than treatment failure, potential attrition bias cannot be excluded and should be considered when interpreting outcome analyses.

Finally, cost considerations and limited availability continue to restrict widespread use of FDG-PET/CT in routine clinical practice, underscoring the need for prospective studies to identify patient subgroups that derive the greatest benefit from this imaging modality.

## 5 Conclusion

FDG PET/CT provides clinically meaningful added value in the diagnostic workup of spinal infections, particularly as a complementary modality to MRI for the detection of spondylodiscitis and the identification of infectious foci. In this context, FDG-PET/CT should be integrated into a multimodal diagnostic framework, e. g. supplemented by targeted evaluation for endocarditis and odontogenic sources of infection. Selective incorporation of FDG-PET/CT into diagnostic algorithms for spinal infections may enhance diagnostic confidence and enable more precise decision-making. Future prospective studies are warranted to better define patient subgroups that derive the greatest benefit from PET-guided diagnostic strategies and to clarify its impact on clinical outcomes.

## Statement of ethics and consent to participate

The presented study meets the ethical standards outlined in the Declaration of Helsinki. Ethics approval was obtained by our local ethics committee, and the favorable vote was registered under the number 2023-324_1-S-SB.

## Availability of data and materials

The datasets used and analyzed during the current study are available from the corresponding author upon reasonable request.

## Contribution

Conception and design: BM, WW.

Acquisition of data: RL, SH, LR.

Analysis and interpretation of data: RL, SN, SI, EU, MW, AKJ.

Manuscript draft: RL, AKJ.

Critical revision for important intellectual content: BM, WW.

Final approval: RL, AKJ, SN, BM, WW.

## Funding

This research did not receive any specific grant from funding agencies in the public, commercial, or not-for-profit sectors.

## Conflict of interest

We, at this moment, declare no conflict of interest.
